# *In Vivo* Effect of Pneumonia on Surfactant Disaturated-Phosphatidylcholine Kinetics in Newborn Infants

**DOI:** 10.1371/journal.pone.0093612

**Published:** 2014-12-31

**Authors:** Maddalena Facco, Matteo Nespeca, Manuela Simonato, Ilena Isak, Giovanna Verlato, Gianluca Ciambra, Chiara Giorgetti, Virgilio P. Carnielli, Paola E. Cogo

**Affiliations:** 1 Department of Women's and Children's Health, University of Padua, Padua, Italy; 2 Neonatal Medicine, Salesi Children's Hospital, Polytechnic University of Marche and University of Ancona, Ancona, Italy; 3 Pediatric Cardiac Anesthesia/Intensive Care Unit, Department of Pediatric Cardiology and Cardiac Surgery, Bambino Gesù Children's Hospital, Rome, Italy; Icahn School of Medicine at Mount Sinai, Argentina

## Abstract

**Background:**

Bacterial pneumonia in newborns often leads to surfactant deficiency or dysfunction, as surfactant is inactivated or its production/turnover impaired. No data are available *in vivo* in humans on the mechanism of surfactant depletion in neonatal pneumonia. We studied the kinetics of surfactant's major component, disaturated-phosphatidylcholine (DSPC), in neonatal pneumonia, and we compared our findings with those obtained from control newborn lungs.

**Methods:**

We studied thirty-one term or near-term newborns (gestational age 39.7±1.7 weeks, birth weight 3185±529 g) requiring mechanical ventilation. Fifteen newborns had pneumonia, while 16 newborns were on mechanical ventilation but had no lung disease. Infants received an intratracheal dose of ^13^C labeled dipalmitoyl-phosphatidylcholine at the study start. We measured the amount and the isotopic enrichment of DSPC-palmitate from serial tracheal aspirates by gas chromatography and gas chromatography-mass spectrometry, respectively, and we calculated the DSPC half-life (HL) and pool size (PS) from the isotopic enrichment curves of surfactant DSPC-palmitate.

**Results:**

The mean DSPC amount obtained from all tracheal aspirates did not differ between the two groups. DSPC HL was 12.7 (6.5–20.2) h and 25.6 (17.9–60.6) h in infants with pneumonia compared with control infants (p = 0.003). DSPC PS was 14.1 (6.6–30.9) mg/kg in infants with pneumonia and 34.1 (25.6–65.0) mg/kg in controls, p = 0.042. Myeloperoxidase (MPO) activity, as a marker of lung inflammation, was 1322 (531–2821) mU/ml of Epithelial Lining Fluid (ELF) and 371(174–1080) mU/ml ELF in infants with pneumonia and in controls, p = 0.047. In infants with pneumonia, DSPC PS and HL significantly and inversely correlated with mean Oxygenation Index (OI) during the study (DSPC PS vs. OI R = −0.710, p = 0.004 and HL vs. OI R = −0.525, p = 0.044, respectively).

**Conclusions:**

We demonstrated for the first time *in vivo* in humans that DSPC HL and PS were markedly impaired in neonatal pneumonia and that they inversely correlated with the degree of respiratory failure.

## Introduction

Pulmonary surfactant plays an important role in the host defense against respiratory infections. Bacterial pneumonia of near-term or term newborns often leads to surfactant deficiency or dysfunction, as surfactant is inactivated, peroxidated, or its secretion is impaired [1]–[3]. Research on the benefits of exogenous surfactant for the treatment of neonatal pneumonia is not well established. Studies on animal models [4]–[6], clinical case reports [7], and non-randomized clinical trials [8]–[10] that included mostly preterm infants, suggested that exogenous surfactant might be useful to infants with bacterial pneumonia. Almost all these studies included infants < 33 weeks gestational age, who received exogenous surfactant for primary surfactant deficient respiratory distress syndrome (RDS) associated with congenital or acquired bacterial pneumonia [11], [12]. Little information is available on term and near-term infants with neonatal pneumonia without RDS. Indirect evidence of a beneficial effect of exogenous surfactant on neonatal pneumonia can be derived from the use of surfactant in infants with meconium aspiration syndrome where surfactant inactivation could be the main mechanism of surfactant deficiency, similar to what is observed in neonatal pneumonia [13]. In these studies, dosing and the means of administration were important aspects of the response to exogenous surfactant [14], [15]. However, a recent Cochrane review underlined the lack of randomized clinical trials (RCTs) supporting or refuting the clinical benefits and safety of exogenous surfactant in neonatal pneumonia [16]. The authors of that review advocated future RCTs on term or near-term infants with proven bacterial pneumonia to test the efficacy of higher or more frequent surfactant doses than those required for replacement therapy in preterm infants with RDS (11). A deeper knowledge of surfactant pharmacokinetics in neonates with bacterial pneumonia not treated with exogenous surfactant would be of great value in designing these trials.

Studies on exogenous surfactant pharmacokinetics have become available only in recent years using DSPC (the most abundant surfactant phospholipid component) labeled with stable isotopes as an indicator of lung surfactant kinetics [17]–[19].

We applied these techniques to evaluate whether alterations of endogenous surfactant kinetics occur in term or near-term neonates with pneumonia and are associated with the severity of the lung disease. This hypothesis, if confirmed, would strengthen the rationale for novel trials with tailored exogenous surfactant doses and/or rates of surfactant administration as treatments for severe neonatal pneumonia.

## Methods

### Patients and study design

We conducted a prospective study in the Neonatal Intensive Care Units of the University of Padua and of the Polytechnic University of Marche, Ancona, Italy. We recruited 31 term newborns on mechanical ventilation for severe pneumonia (15 infants) and for major surgery or neurological failure but with no lung disease (16 infants). The study lasted from January 2008 to December 2011.

Eligible infants were late preterm (35 to 36 weeks completed gestation) or term newborns (37 to 41 weeks completed gestation) up to 28 days of life with diagnosis of pneumonia, defined according to the 2008 CDC/NHSN (Centers for Disease Control and Prevention/National Healthcare Safety Network) criteria for pneumonia in infants ≤1 year of age [20]. These are basically limited to clinically defined pneumonia (PNU1), characterized by: i) Chest X-Ray showing new or progressive or persistent infiltrate, consolidation or cavitation or pneumatoceles; ii) Worsening gas exchange (desaturation or rise in oxygen requirement or rise in ventilation demand); iii) At least 3 of the following: temperature instability with no other recognized cause; leukopenia (<4000 WBC/mm^3^) or leucocytosis (≥15000 WBC/mm^3^) with left shift (≥10% band cells); new onset of purulent sputum, or change in character of sputum, or increased respiratory secretions, or increased suctioning requirements; apnea, tachypnea, nasal flaring with retraction of chest wall or grunting; wheezing, rales or rhonchi; cough; bradycardia (<100 beats/min) or tachycardia (>170 beats/min).

Inclusion criteria were: a) severe respiratory failure (newborns with pneumonia) requiring mechanical ventilation with a Fraction of Inspired Oxygen, (FiO_2_) >0.35 and a Mean Airway Pressure, (MAP) >7 cm H_2_O, and a prediction to be on a ventilator for at least 48 h; b) newborns with no lung disease (controls), requiring mechanical ventilation as a result of major surgery or neurological failure leading to poor airway control; c) the presence of an indwelling arterial line, inserted for close clinical monitoring and gas exchange analysis, required for the severity of respiratory failure. Exclusion criteria were: severe congenital malformations, chromosomal abnormalities, and exogenous surfactant administration at the time of the study.

The protocol was approved by the Ethics Committee of the University of Padua and by the Ethics Committee of the Polytechnic University of Marche, and written informed consent was obtained from both parents of each infant. No external funding sources were granted for this study. Parents were informed that their child was not going to benefit from the study, but that the study results could potentially help other infants with the same clinical condition.

As soon as the mechanical ventilation was started, and after the informed consent was obtained, all study infants received an intratracheal tracer dose (2 mg/kg) of U^13^C-Dipalmitoyl-Phosphatidylcholine (U^13^C-DPPC) mixed with a vehicle dose of 2 to 5 mg/kg of porcine surfactant (Curosurf, Chiesi Pharmaceuticals, Parma, Italy) via a small catheter inserted through the endotracheal tube at the carina level, to trace endogenous pulmonary surfactant. Stable isotope tracers are safe for human studies; they were prepared in a sterile way by the hospital pharmacy and the delivering procedure has been performed at several times in both Units with no side effects [17]–[19], [21]–[25].

We collected tracheal aspirates before the administration of the tracer (t = 0), every 6 h for the first 72 h, and then every 12 h for another 4 days or until extubation. The rate and the way of suctioning is standardized in both units for all ventilated patients; for this study we collected the secretions, that otherwise would have been discarded, in Lukens trap at the pre-set time points [21]. We recorded clinical data, vital and ventilator parameters hourly from our computerized bedside monitoring systems, and we performed arterial blood gas analysis every 6 hours to calculate Oxygenation Index (OI) as ((MAP*FiO_2_/PaO_2_)*100), and PaO_2_/FiO_2_ ratio as indicators of respiratory failure. The rate of monitoring, including arterial gas analysis, were prescribed independently from the study by the attending physician, thus no change was provided in the routine clinical assistance.

### Tracheal aspirate collection and storage

Tracheal aspirates were obtained as previously described [21], [22]. Briefly, after instillation of 1 ml saline (0.9% NaCl) in the endotracheal tube, the neonate was gently hand-bagged and then tracheal secretions were collected through a Lukens trap. The samples were stored at 4°C for no longer than 3 h and brought to a final volume of 2 ml with 0.9% saline. Tracheal aspirates with visible blood were discarded. After gentle vortexing for 1 minute, 300 µl of tracheal aspirate fluid were freeze-thawed three times in liquid nitrogen and centrifuged at 13,000 rpm (2,325×g) for 45 min at 4°C. The supernatant was collected and stored at −20°C for myeloperoxidase activity (MPO) determination. The remaining tracheal aspirate volume was centrifuged at 400×g for 10 min. The supernatant was recovered and stored at −20°C until analysis.

### Analytical methods

Lipids from tracheal aspirates were extracted using Bligh and Dyer's method [26] after addition of the internal standard heptadecanoylphosphatidylcholine. DSPC was isolated from the lipid extract by thin layer chromatography (TLC) [27]. DSPC fatty acids were derivatized as methyl-esters and stored at −20°C. DSPC fatty acid composition was analyzed by gas-chromatography-FID and the ^13^C enrichment of DSPC-palmitate by gas-chromatography-mass spectrometry (GC-MS Agilent, Milan, Italy). Enrichment values were expressed as mole percent excess (MPE).

MPO activity was measured spectrofotometrically using O-dianisidine and hydrogen peroxide. One unit (U) of MPO activity was defined as that degrading 1 µmol of hydrogen peroxide per minute at 25°C [18].

MPO activity and DSPC amounts were corrected by the urea dilution method and expressed as U/ml of Epithelial Lining Fluid (ELF) [28].

### Calculations and statistical analysis

Surfactant DSPC kinetics and pools were calculated from the exponential region of the DSPC ^13^C-enrichment curves of serial tracheal aspirates as previously described in detail [18], [21]. Statistical analysis was performed using PASW Statistics 18.0 for Windows (SPSS Inc., Chicago, IL). Data are expressed as mean ± SD or median (IQR) according to the variable distribution. Comparisons were made by Student t-test and Mann-Whitney test for continuous variables, and by Chi-square for categorical variables. A p≤0.05 was considered as statistically significant. Spearman's correlation analysis was performed to test the dependency of DSPC HL and PS on ventilator and oxygenation parameters.

## Results

We studied 31 term infants: 15 infants with pneumonia and 16 with no lung disease. Clinical characteristics of the two groups were reported in Table 1. Among control infants one had congenital myopathy, 5 had abdominal surgery requiring prolonged sedation and mechanical ventilation and 9 had hypoxic-ischemic encephalopathy with no oxygen dependency and with the need of mechanical ventilation due to neurological failure.

**Table 1:** Clinical characteristics of study patients.

	Newborns with pneumonia (N = 15)	Newborns with no lung disease (N = 16)	p
Gestational Age (wks) (mean ± SD)	39.3±1.2	40.1±1.9	0.239
Birth Weight (g) (mean ± SD)	3197±711	3173±283	0.909
Gender (N°females/N°males)	5/10	6/10	0.553
Infants studied in centre 1 and 2 (N°/N°)	4/11	7/9	0.269
Intubation – study start interval (h) (median(IQR))	41(18–75)	19(10–30)	0.057
Age at study start (days) (median(IQR))	2.3(1.2–3.7)	2.4(1.1–7.5)	0.693
CRP at study start (mg/L) (median(IQR))	26.1(14.5–50.0)	2.0(1.0–3.4)	<0.001
CRP during study (mg/L) (median(IQR))	29.6(13.1–69.8)	7.7(3.3–39.5)	0.022

Ventilator and oxygenation parameters were shown in Table 2. We found significant differences between the two groups in CRP (C-Reactive Protein), MAP, FiO_2_, arterial PaO_2_, OI and PaO_2_/FiO_2_ ratio at the start of the study, according to the study design. The study started 41(18–75) h and 19(10–30) h after intubation in newborns with pneumonia and in the controls, respectively, (p = 0.057). This long time interval from intubation to the study start was necessary to match all the criteria required for the pneumonia diagnosis, and also to leave to the parents a convenient time to give the informed consent (the policy of both institutions is to leave to the parents the time to think about it before giving the consent).

**Table 2:** Ventilator and oxygenation parameters of patients at study start.

	Newborns with pneumonia (N = 15)	Newborns with no lung disease (N = 16)	p
Ventilator style (N° HFOV/N° SIMV)	4/11	0/16	0.043
MAP (cmH_2_O) (median(IQR))	8.2(6.7–12.0)	5.7(4.8–7.2)	0.001
FiO_2_ (mean ± SD)	0.54±0.25	0.22±0.02	<0.001
OI (median(IQR))	5.9(3.3–10.4)	1.8(1.2–2.8)	<0.001
PaO_2_/FiO_2_ (median(IQR))	124(90–238)	334(262–394)	<0.001
Arterial pH (median(IQR))	7.36(7.30–7.41)	7.35(7.31–7.40)	0.908
Arterial pO_2_ (median(IQR))	62.4(58.1–72.6)	82.6(65.3–84.0)	0.014

HFOV: High-Frequency Oscillatory Ventilation; SIMV: Synchronized Intermittent Mandatory Ventilation.

Survival to discharge was similar between the two groups (14 out of 15 in the pneumonia group, and 14 out of 16 in the control group, p = 0.525). No newborn was treated with Nitric Oxide and none developed oxygen dependency at 28 days of life.

MPO activity was significantly higher in the tracheal aspirates of newborns with pneumonia compared to those with no lung disease (1322 (531–2821) mU/ml ELF and 371 (174–1080) mU/ml ELF, p = 0.047, respectively) [28].

Mean DSPC concentrations in tracheal aspirates during the study period were not significantly different in the two study groups, being 1.2 (0.4–7.5) mg/ml ELF in the pneumonia group and 2.4 (0.7–3.9) mg/ml ELF in the control group, p = 0.790.

**Figure 1:**
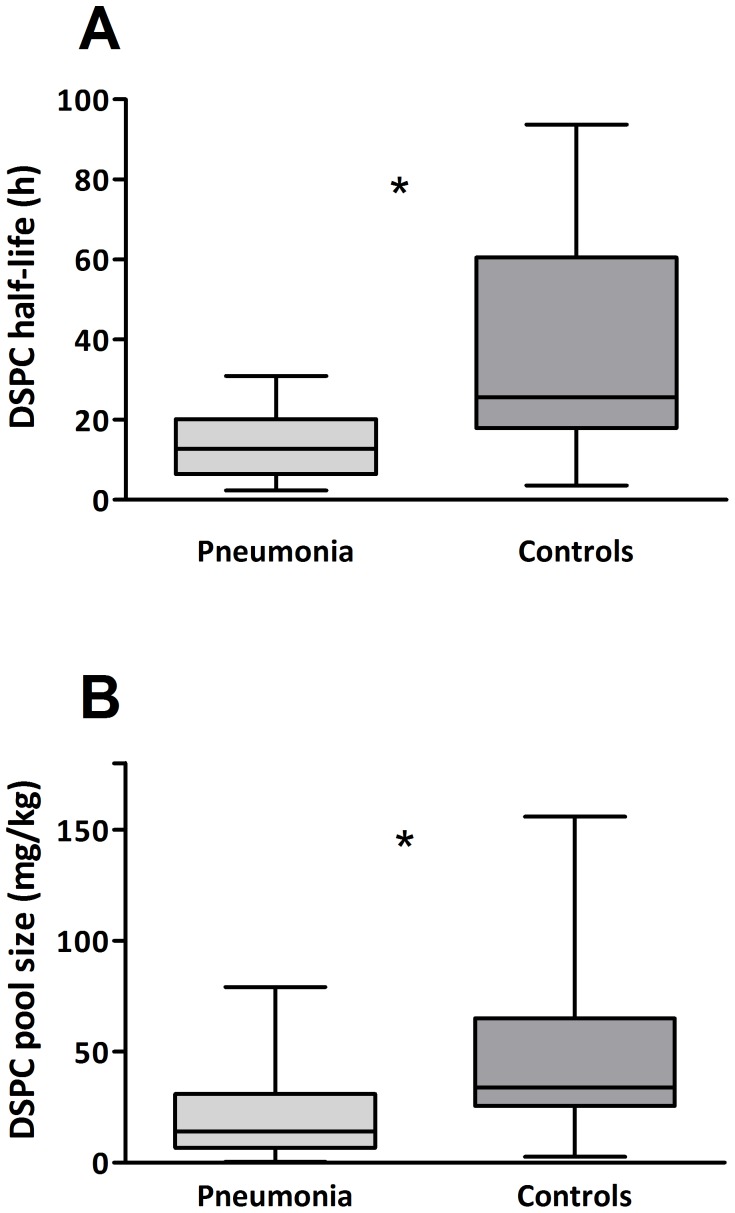
DSPC half-life (HL) and pool size (PS) in the two study groups. Panel A: Median DSPC HL in the two groups. DSPC HL was significantly shorter in newborns with pneumonia (light grey box) compared with newborns with no lung disease (grey box). Data are expressed as median (IQR). Panel B: Median DSPC PS in the two groups. DSPC PS was significantly lower in newborns with pneumonia (light grey box) compared with newborns with no lung disease (grey box). Data are expressed as median (IQR).

Surfactant DSPC kinetics could be calculated in all study patients. Median DSPC HL was significantly shorter in the pneumonia group compared to the median DSPC HL in the control group (12.7 (6.5–20.2) h and 25.6 (17.9–60.6) h, p = 0.003, respectively, Fig. 1 panel A). Similarly, median DSPC PS was found to be significantly lower in newborns with pneumonia compared to newborns with no lung disease (14.1 (6.6–30.9) mg/kg body weight and 34.1 (25.6–65.0) mg/kg body weight, p = 0.042, respectively, Fig. 1 panel B).

Four out of 15 newborns with pneumonia required High Frequency Oscillatory Ventilation (HFOV), while all newborns in the control group were ventilated by Synchronized Intermittent Mandatory Ventilation (SIMV). Median DSPC HL was 9.7 (6.9–12.3) h in newborns on HFOV and 18.4 (5.2–20.8) h in newborns on SIMV, (p = 0.296). Median DSPC PS was markedly lower in the 4 infants who required HFOV compared to those ventilated with SIMV (3.1 (1.2–7.8) mg/kg body weight and 29.7 (9.3–31.7) mg/kg body weight, respectively, p = 0.036).

In the pneumonia group a significant correlation was found between DSPC PS and mean OI during the study (R = −0.710, p = 0.004) and also between DSPC HL and mean OI during the study (R = −0.525, p = 0.044).

## Discussion

In this study, we measured for the first time surfactant DSPC kinetics in newborns with pneumonia and we found that endogenous surfactant DSPC HL and PS were markedly reduced in infants with pneumonia not treated with exogenous surfactant. We used a state of the art method, based on safe stable isotope markers [19], [23], which is applicable *in vivo* to intubated patients from whom sequential tracheal aspirates can be collected as part of the routine clinical care. We chose to assess the endogenous DSPC kinetics. Therefore we administered 2 to 5 mg/kg surfactant mixed with ^13^C DPPC, which cannot be considered a surfactant treatment dose. The advantage of using a stable isotope technique lies in the fact that kinetic data are more informative than DSPC concentrations. Measuring DSPC amounts from tracheal aspirates with or without a correction for dilution [28] can be deceiving because of the highly variable contribution of phospholipids from inflammatory cells. In this light, the mean amount of DSPC recovered was not different between the study groups.

The major finding of this study is that DSPC HL was significantly shorter in pneumonia than in controls. Interestingly, these HL values were similar to those reported by our group in the most severe RDS patients who required repeated doses of exogenous surfactant to overcome their respiratory failure [24], suggesting that the DSPC turnover in term or near term pneumonia is at least as fast as in the most severe cases of RDS.

However the efficacy of exogenous surfactant on neonatal pneumonia is still to be determined [16]. Only two non-randomized studies are available on the clinical outcome of neonatal pneumonia treated with exogenous surfactant. The first one included only preterm infants and found no difference in clinical outcome in infants with pneumonia compared with RDS [29]. The second one evaluated preterm and term infants with streptococcus B pneumonia treated with natural surfactant at doses of 100 or 200 mg/kg and found a slower clinical improvement in the pneumonia group compared to RDS [10].

The shorter DSPC HL likely led in infants with pneumonia to a significant reduction of surfactant PS, which was about one third of that measured in newborns with healthy lungs (controls). This is a novel result. Hallman *et al.* [30] and Griese *et al.* [31] reported PS estimates in preterm newborns with RDS of about 16 mg/kg. Values ranging from 1 to 15 mg/kg were found in preterm infants with RDS before exogenous surfactant [19], [21], [24], [25]; in adults with ARDS we found that surfactant PS was markedly and significantly lower than in controls [23]. Since pneumonia shares a certain pathophysiology with ARDS [32], it is not surprising to find similar alterations of surfactant pools, mediated by alveolar-capillary leaks of proteinaceous edema and inflammatory cells that contribute to surfactant inactivation and catabolism [3], [33], [34]. The significant increase of MPO activity that we found in infants with pneumonia supports this hypothesis. Whether exogenous surfactant can counteract the lack of endogenous surfactant pool in the acute phase of ARDS in infants is still debated. The only blinded, randomized, controlled study performed in ALI/ARDS pediatric patients who received natural surfactant (calfactant) relative to placebo (77 surfactant treated and 75 placebo) reported an immediate improvements in oxygenation and a significant survival advantage for patients receiving surfactant treatment.(33).

Four study infants with pneumonia received HFOV as rescue treatment for respiratory failure not responding to conventional ventilation. Interestingly these infants, who had the most severe degree of respiratory failure, had the most severe impairment of DSPC kinetics, with a significantly lower DSPC PS compared with controls. We hypothesize that the use of HFOV may not be responsible for the DSPC kinetic alterations, since the open lung concept, as an alternative ventilatory strategy, is reported to be a protective strategy for lung injury [35]–[37].

We also found that mean OI significantly correlated with DSPC PS and DSPC HL. Conversely, mean OI did not correlate with gestational age, the age at the start of the study, duration of mechanical ventilation and MPO activity. It will be difficult to ascertain whether FiO_2_ and mechanical ventilation play significant and independent roles in surfactant kinetics, or are proxies for respiratory severity. Mechanical ventilation and oxygen exposure may act as injury promoters in critically ill patients [38], inducing cytokine upregulation in both healthy and injured lungs [39]. Animal studies could help address this issue.

A limitation of our study is that the control infants were on mechanical ventilation for neuromuscular causes, prolonged sedation or neurological impairment. Although we acknowledge that the gold standard “controls” would have been infants spontaneously breathing room air, our study provides the best estimation of endogenous surfactant kinetics that can be obtained in humans with healthy lungs.

To our knowledge, this is the first report in neonatal pneumonia to show decreased surfactant DSPC PS and HL associated with disease severity in term or near-term newborn not treated with exogenous surfactant. The effect of exogenous surfactant administration on the endogenous surfactant kinetics is still unknown.

As we found comparable DSPC kinetic alterations in infants with the most severe RDS cases, who required multiple surfactant administrations [24], we speculate that higher doses of exogenous surfactant or shorter redosing time intervals than those used in neonatal RDS may be more appropriate for the treatment of the most severe cases of neonatal pneumonia.

## Conclusions

In this study we reported the endogenous surfactant kinetics of late preterm and term newborns with pneumonia. DSPC HL and PS were markedly impaired in newborns with pneumonia. DSPC HL and PS were associated with oxygen requirement, which is a proxy for severity of lung disease. Further studies are required to confirm whether exogenous surfactant therapy could prolong DSPC HL, increase DSPC PS, and improve clinical outcome in neonatal pneumonia.
